# Effects of Dietary Vitamin B6 Restriction on Hepatic Gene Expression Profile of Non-Obese and Obese Mice

**DOI:** 10.3390/nu12123821

**Published:** 2020-12-14

**Authors:** Hyun-Jee Um, Je Won Ko, Sae Bom Won, Young Hye Kwon

**Affiliations:** 1 Department of Food and Nutrition, Seoul National University, Seoul 08826, Korea; umhj1209@snu.ac.kr (H.-J.U.); kgrami@snu.ac.kr (J.W.K.); 2 Research Institute of Human Ecology, Seoul National University, Seoul 08826, Korea; 3 Department of Human Nutrition and Food Science, Chungwoon University, Hongseong, Chungnam 32244, Korea; newspring@chungwoon.ac.kr

**Keywords:** amino acid metabolism, dietary vitamin B6 restriction, high-fat diet, immunity, mice, sterol metabolism, transcriptome

## Abstract

Although vitamin B6 is contained in various foods, its deficiency is one of the most common micronutrient deficiencies worldwide. Furthermore, patients with obesity and cardiovascular disease are more likely to have suboptimal vitamin B6 status than healthy people. Therefore, we investigated the effects of dietary vitamin B6 restriction on hepatic gene expression and function in obese mice. C57BL/6J male mice were fed a low-fat (LF) or high-fat (HF) diet in combination with sufficient (7 mg pyridoxine/kg diet) or insufficient (1 mg) amounts of vitamin B6 for 16 weeks. Analysis of microarray data revealed that expressions of 4000 genes were significantly altered by the experimental diets (LF7, LF1, HF7, and HF1). The effects of dietary fat content on gene expressions were markedly greater than vitamin B6 content. Only three differentially expressed genes (DEGs) were overlapped between the LF1/LF7 and HF1/HF7 comparison. In the LF1/LF7 comparison, 54 upregulated DEGs were enriched in gene ontology (GO) terms associated with the sterol metabolic process and 54 downregulated DEGs were enriched in GO terms associated with immune response. In HF1/HF7 comparison, 26 upregulated DEGs were enriched in GO terms associated with amino acid catabolic process. High-fat consumption downregulated gene expressions associated with vitamin B6-dependent pathways. In conclusion, our data suggest that obesity may differentially regulate vitamin B6-associated metabolic pathways in the body.

## 1. Introduction

Epidemiological studies have reported that an insufficient intake of vitamin B6 and low plasma concentrations of pyridoxal 5′-phosphate (PLP), a metabolically active form of vitamin B6, are associated with diabetes, neurological disorders, cardiovascular disease, and cancer [1,2,3,4]. Furthermore, vitamin B6 deficiency has been shown to alter both cell-mediated and humoral immunity [5,6]. PLP serves as a coenzyme of numerous enzymatic reactions in carbohydrate, lipid, and amino acid metabolism through decarboxylation, transamination, racemization, elimination, and β-group interconversion [7]. In particular, PLP plays a pivotal role in maintaining normal homocysteine levels by regulating the activities of PLP-dependent enzymes in transsulfuration and transmethylation pathways. Additionally, several studies have reported anti-oxidative [8] and anti-inflammatory [9] effects of vitamin B6.

Since vitamin B6 is found in various foods, dietary vitamin B6 inadequacy is difficult to find among people who eat regular diets. However, vitamin B6 insufficiency is common [10,11,12,13] and could be aggravated by a food-derived anti-pyridoxine factor [14]. Plasma PLP concentrations less than 20 nmol/L indicates deficiency and less than 30 nmol/L indicates marginal deficiency or insufficiency in humans [11,15].

In animal studies of marginal deficiency induced by insufficient vitamin B6 supply, plasma concentrations of amino acids and lipophilic metabolites, such as *N*-acyl amino acids and bile acids, were significantly changed [14,16,17]. Similarly, plasma concentrations of several amino acids and their metabolites, including glycine, asparagine, glutamine, cystathionine, and glutathione, were significantly changed in humans with marginal vitamin B6 deficiency [18,19,20]. However, little is known about the effects of marginal vitamin B6 deficiency on the gene expression profile in the liver. Furthermore, most studies on vitamin B6 deficiency have been conducted on healthy individuals or animals fed a control diet. In this study, we compared the effect of dietary vitamin B6 restriction on hepatic transcriptome between non-obese and high-fat fed obese mice to investigate whether obesity may differentially regulate vitamin B6-associated metabolic pathways in the body.

## 2. Materials and Methods

### 2.1. Animals and Diets

Four-week old C57BL/6J male mice were purchased from Raon Bio (Yongin, Korea) and housed in a temperature (22 ± 3 °C) and humidity (50 ± 10%) controlled room with a 12 h light–dark cycle. After 12 days of acclimation [5 days with a chow diet and 7 days with a control low-fat (12% kcal from fat) diet with 7 mg pyridoxine hydrochloride (PN)/kg diet], mice were randomly divided into the four groups (*n* = 8–9) and fed an LF7 (low-fat diet with 7 mg PN/kg diet), LF1 (low-fat diet with 1 mg PN/kg diet), HF7 [high-fat (45% kcal from fat) diet with 7 mg PN/kg diet], or HF1 (high-fat diet with 1 mg PN/kg diet) diet. The experimental diets were provided ad libitum for 16 weeks. The composition of the diets is shown in Supplementary Materials Table S1. The recommended amount of PN is a 7 mg/kg diet according to the AIN-93 formulation [21] and a 1 mg PN/kg diet has been reported as the minimum level to prevent a growth retardation [22]. At the end of the experiments, the animals were fasted for 12 h and sacrificed after intraperitoneal injection of 30 mg/kg Zoletil (Virbac, Carros, France) and 10 mg/kg Rompun (Bayer Korea, Seoul, Korea). Blood samples were collected by cardiac puncture. To obtain serum, blood was left at room temperature for 1 h and centrifuged at 3000 rpm, 4 °C for 20 min. The organs were excised, frozen in liquid nitrogen, and stored at −80 °C until analysis. All animal experiments were approved by Institute of Laboratory Animals Resources of Seoul National University (SNU-161111-1-2), and conducted in accordance with the guidelines of Institutional Animal Care and Use Committee of Seoul National University.

### 2.2. Serum and Hepatic Biochemical Analysis

Serum glucose, triglyceride (TG), total cholesterol, and glutamate-pyruvate transaminase (GPT) concentrations were analyzed using commercial colorimetric assay kits (Asan Pharmaceutical Co., Seoul, Korea) according to the manufacturer’s protocol. After extraction of hepatic total lipids [23], cholesterol concentrations were determined using the same commercial kit. Hepatic TG and total bile acid concentrations were determined by thin-layer chromatography [24] and a total bile assay reagent kit (Gentaur, San Jose, CA, USA), respectively. Hepatic total homocysteine concentrations were measured using high-performance liquid chromatography, as previously described [25].

### 2.3. Tissue Histologic Examination

The dissected liver tissues were fixed with formalin, embedded in paraffin, sectioned into 4 μm-thick sections, and stained with hematoxylin and eosin (H&E) for histological analysis. The morphology was examined under an Olympus BX50 microscope using a DP-72 digital camera (Olympus, Tokyo, Japan) and captured using the Image-Pro Plus program (Media Cybernetics Inc., Rockville, MD, USA).

### 2.4. Microarray Analysis

Total hepatic RNA was extracted using RNAiso Plus (Takara Bio Inc., Shiga, Japan) and RNA purity and integrity were confirmed by Agilent 2100 Bioanalyzer (Agilent Technologies, Santa Clara, CA, USA). Transcriptome analysis was performed with Affymetrix Mouse Clariom S arrays (Thermo Fisher Scientific, Waltham, MA, USA) as described previously [26]. To identify differentially expressed genes (DEGs) among groups, statistical significance of the expression data was determined using one-way analysis of variance (ANOVA, *p* < 0.05). DEGs between two groups were selected based on the cut-off criteria (|fold change| ≥ 1.5 and *p* < 0.05). Functional analysis of DEGs was performed using the Gene Ontology (GO) database, Kyoto Encyclopedia of Genes and Genomes (KEGG) pathway, and WikiPathways. False discovery rate was corrected by *p*-value using the Benjamini–Hochberg algorithm. Heatmaps and volcano plots were constructed using the PermutMatrix software and R package, respectively.

### 2.5. Statistical Analysis

Experimental results except for microarray data were analyzed using SPSS software (version 23; IBM SPSS Inc., Armonk, NY, USA). Data are expressed as the mean ± SEM and were analyzed by one-way ANOVA followed by Duncan’s multiple range test. Differences with *p* < 0.05 were considered statistically significant.

## 3. Results

### 3.1. Effects of Dietary Vitamin B6 Restriction on Serum and Hepatic Biochemical Parameters of Mice

**Figure 1:**
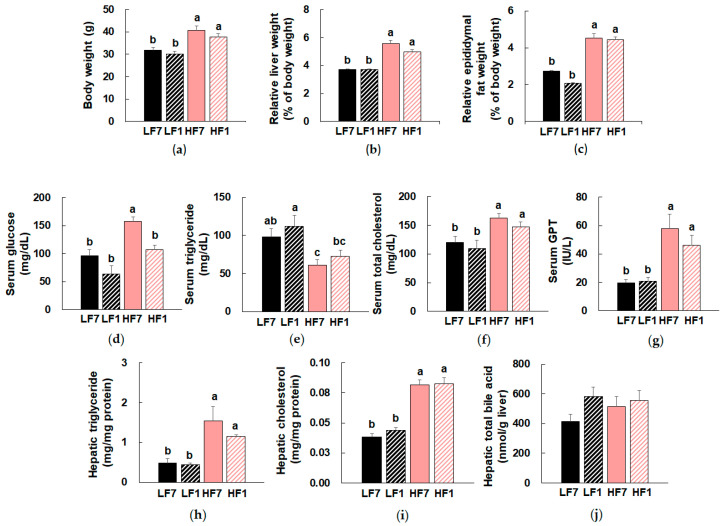
Effects of dietary vitamin B6 restriction on anthropometric and biochemical parameters of mice. (**a**) Body weight, (**b**) relative liver weight, and (**c**) relative epididymal fat weight. Serum (**d**) glucose, (**e**) triglyceride, (**f**) total cholesterol, and (**g**) GPT levels. Hepatic (**h**) triglyceride, (**i**) cholesterol, and (**j**) total bile acid levels. Data are expressed as means ± SEMs (*n* = 7–9). Means with the same letter are not significantly different from each other (one-way ANOVA, *p* < 0.05). LF7, a low-fat diet with 7 mg pyridoxine hydrochloride (PN)/kg diet; LF1, a low-fat diet with 1 mg PN/kg diet; HF7, a high-fat diet with 7 mg PN/kg diet; HF1, a high-fat diet with 1 mg PN/kg diet.

Consumption of a high-fat diet significantly increased body weight in both vitamin B6-sufficient and -deficient groups (Figure 1a). Relative weights of liver and epididymal fat were also significantly increased by high-fat feeding (Figure 1b,c). Both body weight and relative organ weights were not significantly changed by dietary vitamin B6 amount.

High-fat feeding significantly increased serum glucose concentrations, which were decreased by dietary vitamin B6 restriction (Figure 1d). Serum triglyceride concentrations were significantly reduced by high-fat feeding, which tended to increase in mice fed a vitamin B6-restricted diet (Figure 1e). No significant effect of dietary vitamin B6 restriction was observed in serum cholesterol, serum GPT, hepatic triglyceride, and hepatic cholesterol levels, which were significantly higher in HF-fed mice compared to LF-fed mice (Figure 1f–i, Supplementary Materials Figure S1). Hepatic bile acid levels were not significantly different between the groups (Figure 1j). Previous studies have reported that mice with hepatic steatosis exhibited increased free fatty acid uptake and TG synthesis [27]. Furthermore, stimulation of lipoprotein lipase activity has been shown to be associated with reduced serum triglyceride concentrations in subjects with hepatic steatosis [28].

### 3.2. Effects of Dietary Vitamin B6 Restriction on Hepatic Transcriptome of Mice

**Figure 2:**
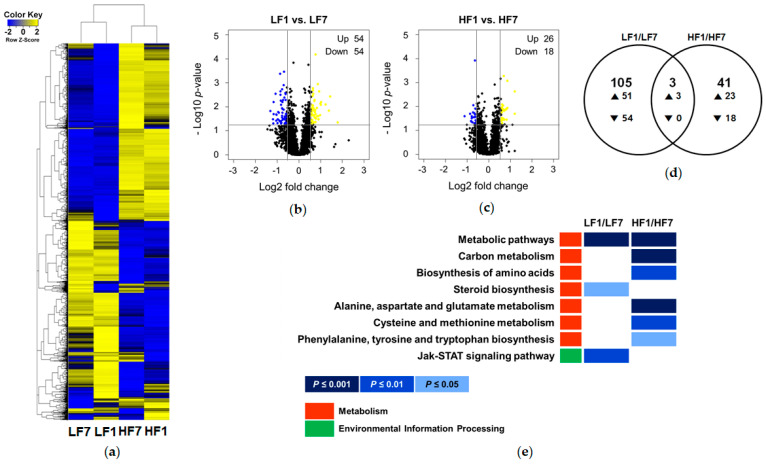
Effects of dietary vitamin B6 restriction on hepatic gene expression profile. (**a**) A heatmap overview of the 2-way hierarchical clustering analysis of differentially expressed gene (DEG) (*n* = 3) identified by one-way ANOVA (*p* < 0.05). Volcano plots of the DEGs in (**b**) LF1/LF7 and (**c**) HF1/HF7 comparisons. Yellow dots represent the upregulated genes and blue dots represent the downregulated genes by the criteria of *p* < 0.05 and |fold change| ≥ 1.5. (**d**) Venn diagrams of DEG sets. (**e**) Enriched Kyoto Encyclopedia of Genes and Genomes (KEGG) pathways of the DEGs of LF1/LF7 and HF1/HF7 comparisons. LF7, a low-fat diet with 7 mg pyridoxine hydrochloride (PN)/kg diet; LF1, a low-fat diet with 1 mg PN/kg diet; HF7, a high-fat diet with 7 mg PN/kg diet; HF1, a high-fat diet with 1 mg PN/kg diet.

Global gene expression was analyzed to identify signatures in hepatic genes related to experimental diets. Based on microarray analysis, expressions of 4000 genes were significantly altered and were largely dependent on fat content rather than vitamin B6 content in the diet (Figure 2a). Figure 2b,c show volcano plots of DEGs altered by vitamin B6 amount in mice fed a low-fat and high-fat diet, respectively. Among 108 DEGs in the LF1/LF7 comparison and 44 DEGs in HF1/HF7 comparison, Venn diagram shows only three overlapping DEGs, *Asl*, *Nnmt*, and *Sds* (Figure 2d). All of these genes encode PLP-binding proteins that are involved in amino acid metabolism, including the urea cycle, amino acid methylation, and serine metabolism, respectively.

Furthermore, we found the distinct effects of dietary vitamin B6 restriction in mice fed either LF or HF. According to the KEGG enrichment heatmap, “Jak-STAT signaling pathway” and “steroid biosynthesis”, were significantly associated with DEGs in the LF1/LF7 comparison (Figure 2e). On the other hand, KEGG pathways, such as “carbon metabolism”, “biosynthesis of amino acids”, “alanine, aspartate and glutamate metabolism”, “cysteine and methionine metabolism”, and “phenylalanine, tyrosine and tryptophan biosynthesis” were significantly associated with DEGs in the HF1/HF7 comparison.

### 3.3. Effects of Dietary Vitamin B6 Restriction on Hepatic Transcriptome of Mice Fed a Low-Fat Diet

**Figure 3:**
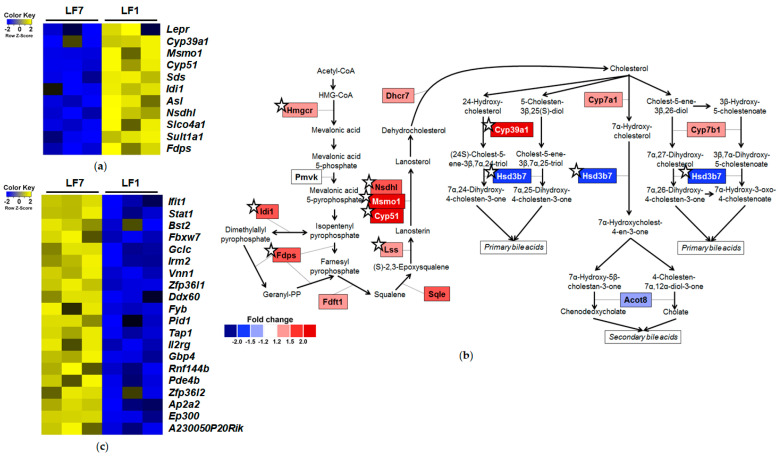
Differentially expressed genes (DEGs) of LF1/LF7 comparison. (**a**) Heatmap of upregulated DEGs enriched in top 10 gene ontology (GO) terms. (**b**) Expression profile of genes involved in cholesterol and bile acid metabolism (modified from Kyoto Encyclopedia of Genes and Genomes pathway and WikiPathway). Significant changes are shown with ☆. (**c**) Heatmap of downregulated DEGs enriched in top 10 GO terms. LF7, a low-fat diet with 7 mg pyridoxine hydrochloride (PN)/kg diet; LF1, a low-fat diet with 1 mg PN/kg diet; HF7, a high-fat diet with 7 mg PN/kg diet; HF1, a high-fat diet with 1 mg PN/kg diet.

Gene functional analysis revealed that 54 upregulated DEGs in the LF1/LF7 comparison were enriched in GO terms in the biological process (BP) category, including “sterol biosynthetic process”, “secondary alcohol biosynthetic process”, and “organic hydroxy compound metabolic process”. The top 10 enriched GO terms are described in Supplementary Materials Table S2. Expression levels of 11 DEGs enriched in the top 10 GO terms are visualized in the heatmap (Figure 3a). We also identified DEGs involved in cholesterol metabolism based on the Wikipathway and KEGG pathway (Figure 3b). Expression levels of *Hmgcr, Idi1, Fdps, Lss, Cyp51, Msmo1, Nsdhl,* and *Cyp39a1* were significantly higher, whereas those of the *Hsd3b7* gene were significantly lower in the LF1 group compared to the LF7 group.

We observed the enriched BP terms related to immune responses, including “regulation of viral process”, “response to cytokine”, “response to interferon-gamma”, and “innate immune response” among the significantly enriched GO terms of 54 downregulated DEGs (Supplementary Materials, Table S3). Expression levels of 20 DEGs enriched in the top 10 GO terms are visualized in the heatmap (Figure 3c).

### 3.4. Effects of Dietary Vitamin B6 Restriction on Hepatic Transcriptome of Mice Fed a High-Fat Diet

**Figure 4:**
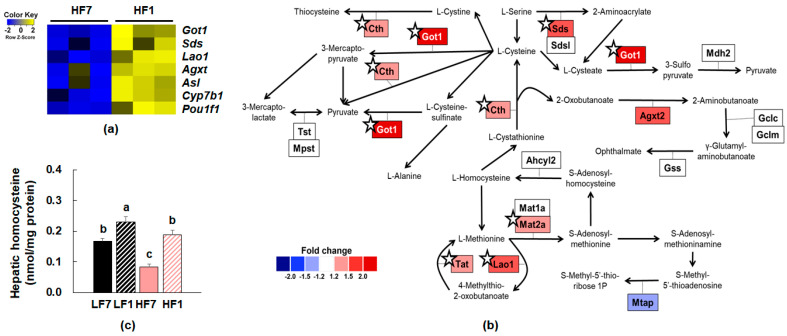
Differentially expressed genes (DEGs) of HF1/HF7 comparison. (**a**) Heatmap of upregulated DEGs enriched in top 10 gene ontology terms. (**b**) Expression profile of genes involved in cysteine and methionine pathway (modified from Kyoto Encyclopedia of Genes and Genomes pathway and WikiPathway). Significant changes are shown with ☆. (**c**) Hepatic homocysteine concentrations (*n* = 7–9). Data are expressed as means ± SEMs. Means with the same letter are not significantly different from each other (one-way ANOVA, *p* < 0.05). LF7, a low-fat diet with 7 mg pyridoxine hydrochloride (PN)/kg diet; LF1, a low-fat diet with 1 mg PN/kg diet; HF7, a high-fat diet with 7 mg PN/kg diet; HF1, a high-fat diet with 1 mg PN/kg diet.

Using functional enrichment analysis of 26 upregulated DEGs in the HF1/HF7 comparison, we identified GO terms in the BP category associated with amino acid and organic acid metabolism, including “cellular amino acid catabolic process”, “alpha-amino acid metabolic process”, and “carboxylic acid catabolic process”. Furthermore, “pyridoxal phosphate binding”, a GO term in molecular function category, was annotated. The top 10 enriched GO terms were described in Supplementary Materials, Table S4. Expression levels of seven genes enriched in the top 10 GO terms are visualized in the heatmap (Figure 4a).

Expression profile of significant genes (one-way ANOVA) enriched in KEGG “cysteine and methionine metabolism” pathway is shown in Figure 4b. Among these genes, mRNA levels of *Cth*, *Got1*, *Lao1*, *Mat2a*, *Sds*, and *Tat*, were significantly upregulated in the HF1 group compared to the HF7 group (Figure 4b). In addition, consumption of a vitamin B6-restricted diet increased the hepatic homocysteine concentration in both LF and HF-fed mice (Figure 4c). Positive associations were observed between hepatic homocysteine levels and mRNA levels of *Cth* (*p* = 0.012), *Lao1* (*p* = 0.005), and *Mat2a* (*p* = 0.042) in high-fat fed mice.

There were no significantly enriched GO terms observed in the enrichment analysis of 18 downregulated DEGs.

### 3.5. Effects of High-Fat Diet on Hepatic Vitamin B6 Metabolism and Function

**Figure 5:**
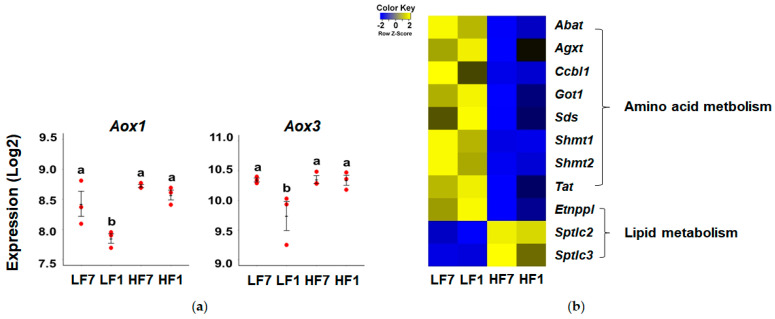
Effects of high-fat diet on hepatic vitamin B6 metabolism and function. (**a**) Hepatic mRNA levels of *Aox1* and *Aox3* were analyzed by microarray. Data are expressed as means ± SEMs (*n* = 3). Means with the same letter are not significantly different from each other (one-way ANOVA, *p* < 0.05). (**b**) Heatmap of differentially expressed genes enriched in gene ontology term “pyridoxal phosphate binding”. LF7, a low-fat diet with 7 mg pyridoxine hydrochloride (PN)/kg diet; LF1, a low-fat diet with 1 mg PN/kg diet; HF7, a high-fat diet with 7 mg PN/kg diet; HF1, a high-fat diet with 1 mg PN/kg diet.

We examined the expression of genes involved in vitamin B6 metabolism. Expression levels of *Pdxk*, *Pdxp*, and *Pnpo*, which convert B6 vitamers to each other, were not significantly different among the groups. On the other hand, expression levels of *Aox1* and *Aox3*, which convert pyridoxal to 4-pyridoxate, a form excreted in urine, were significantly lower in the LF1 group compared to the other groups (Figure 5a). Significant differences were not observed in *Aox1* and *Aox3* mRNA levels of mice fed a high-fat diet in response to dietary restriction of vitamin B6.

In addition, we observed the overall down-regulation of PLP-dependent enzyme-encoding genes in response to high-fat consumption (Figure 5b). Among 11 DEGs enriched in the GO term “pyridoxal phosphate binding”, expression of *Sptlc2* and *Sptlc3* genes involved in lipid metabolism were upregulated and the remaining nine DEGs were downregulated in the high-fat groups compared to the low-fat groups, implicating the overall downregulation of pathways involving PLP as a cofactor in response to a high fat consumption.

## 4. Discussion

Many epidemiological studies indicate that obesity, type 2 diabetes, and cardiovascular disease patients have poorer vitamin B6 status than healthy subjects, implicating the important role of vitamin B6 in the regulation of metabolism [1,2,3]. Furthermore, high intake of vitamin B6 is associated with lower risks of various cancers [29]. Here, we report that high-fat diet-induced obesity differentially regulates vitamin B6-mediated metabolic pathways in the liver based on gene expression profiles of mice fed either a sufficient or restricted vitamin B6 diet. Although several studies have reported that changes in metabolites in marginal vitamin B6 deficiency model, few studies have performed gene expression profiling in this setting. To the best of our knowledge, this study is the first to determine the hepatic gene expression profile in mice fed a vitamin B6-restricted diet.

In comparison of the hepatic gene expression profile between LF1 and LF7 groups, we observed upregulation of genes involved in cholesterol synthesis and downregulation of genes involved in immune response. In previous studies, the effects of vitamin B6 deficiency on cholesterol accumulation were not consistent [30,31,32,33]. Here, we observed that expression levels of several genes involved in cholesterol synthesis were significantly higher in the LF1 group compared to the LF7 group without concomitant changes in serum and hepatic cholesterol concentrations, indicating that dietary vitamin B6 restriction may increase a conversion of cholesterol into bile acid. Accordingly, plasma concentrations of bile acids, including glycocholic acid, glycoursodeoxycholic acid, and murocholic acid, were increased in moderately vitamin B6-deficient rats [17]. Although hepatic total bile acid levels tended to be higher in the LF1 group compared to the LF7 group, this trend did not reach statistical significance. Unlike previous studies using an absolute deficiency of vitamin B6 model [34,35], dietary vitamin B6 restriction did not induce hepatic steatosis.

As shown in the present study in mice fed a low-fat diet, vitamin B6 deficiency is shown to reduce antibody production and to inhibit lymphocyte proliferation, leading to impaired immune responses [5,6,36]. Conversely, there were no significantly enriched GO terms associated with immunity between HF1 and HF7. Because the immune response is highly associated with high-fat feeding, consumption of a high-fat diet may mask the suppressive effect of vitamin B6 on immune response. Indeed, DEGs in the HF7/LF7 comparison were significantly enriched in GO terms, including “immune system process”, “immune response”, and “inflammatory response”.

Consumption of a low vitamin B6 diet altered plasma concentrations of amino acids and their metabolites in human studies [20]. We observed that several amino acid metabolic pathways were significantly regulated by dietary vitamin B6 restriction, especially in mice fed a high-fat diet. Particularly, sulfur amino acid metabolism was significantly altered by vitamin B6 insufficiency in mice fed a high-fat diet. In previous studies, concentrations of cystathionine, a major metabolite in the transsulfuration pathway, were increased in humans and animals with mild and complete vitamin B6 deficiencies [19,20,37]. Interestingly, we observed that consumption of high-fat diet exhibited more apparent regulatory effects on expression of amino acid metabolizing enzymes requiring PLP than consumption of a vitamin-B6-restricted diet. Although we did not measure concentrations of amino acids or amino acid metabolites, these results indicate that vitamin B6 deficiency could play important roles in the regulation of amino acid metabolism in subjects with metabolic syndrome.

There are several possible ways by which vitamin B6 levels regulate gene expression in the present study. First, vitamin B6 has been shown to regulate the functions of steroid hormones, including glucocorticoids, androgen, progesterone, and estrogen by modulating steroid hormone-mediated gene expressions [38]. Second, vitamin B6 level regulates homocysteine levels, resulting in alterations in the cellular SAM:SAH ratio and DNA methyltransferase activity. Accordingly, we observed that hepatic homocysteine concentrations were significantly increased by vitamin B6 deficiency. A previous study reported that PLP deficiency resulted in chromosome breaks and rearrangements in Drosophila, which were increased in the presence of sucrose and its precursors [39].

## 5. Conclusions

Although effects of dietary vitamin B6 absence on regulation of amino acid metabolism have been actively conducted, the involved molecular mechanisms of vitamin B6 are not clearly known. In the present study, we observed functional effects of dietary vitamin B6 restriction on cholesterol metabolism and immune response in mice fed a low-fat diet. On the other hand, functional effects of dietary vitamin B6 restriction on amino acid metabolism were observed in mice fed a high-fat diet by hepatic gene expression profile analysis. These results provide evidence of the distinct role of a high-fat diet in alteration of PLP-associated pathways in the liver of mice in response to dietary vitamin B6 restriction. Because PLP is a cofactor of various enzymes involved in neurotransmitter metabolism, further research is warranted to explore the effects of vitamin B6 insufficiency on brain function.

## Supplementary Materials

The following are available online at https://www.mdpi.com/2072-6643/12/12/3821/s1, Table S1: Composition of experimental diet, Table S2: Top 10 enriched GO terms of upregulated DEGs in LF1/LF7, Table S3: Top 10 enriched GO terms of downregulated DEGs in LF1/LF7, Table S4: Top 10 enriched GO terms of upregulated DEGs in HF1/HF7, Figure S1: Representative hematoxylin and eosin staining of the liver.
