# Supplementary material for: Effects of Dietary Vitamin B6 Restriction on Hepatic Gene Expression Profile of Non-Obese and Obese Mice
Source: Nutrients. 2020 Dec 14;12(12):3821. doi: 10.3390/nu12123821 (PMC7765059; doi:10.3390/nu12123821)
Supplement: Supplementary file 1 [file nutrients-12-03821-s001.pdf]

Supplementary Materials

Table S1. Composition of experimental diet

Ingredient (g)	Diet			
	LF7	LF1	HF7	HF1
Casein	200.0	200.0	200.0	200.0
L-Cystine	3.0	3.0	3.0	3.0
Corn starch	417.5	417.5	117.5	117.5
Maltodextrin	132.0	132.0	132.0	132.0
Sucrose	100.0	100.0	200.0	200.0
Lard	-	-	187.5	187.5
Soybean oil	50.0	50.0	50.0	50.0
Cholesterol	-	-	12.5	12.5
Cellulose	50.0	50.0	50.0	50.0
Mineral mix	35.0	35.0	35.0	35.0
Vitamin mix[1]	10.0	-	10.0	-
Pyridoxine-free vitamin mix[2]	-	10.0	-	10.0
Pyridoxine hydrochloride	-	0.001	-	0.001
Choline bitrate	2.5	2.5	2.5	2.5
Total	1000.0	1000.0	1000.0	1000.0

[1]AIN-93-VX vitamin mix

[2]AIN-93-VX vitamin mix (without pyridoxine-HCl)

LF7, a low-fat diet with pyridoxine hydrochloride (PN) 7 mg/kg diet; LF1, a low-fat diet with PN 1 mg/kg diet; HF7, a high-fat diet with PN 7 mg/kg diet; HF1, a high-fat diet with PN 1 mg/kg diet

Table S2. Top 10 enriched GO terms of upregulated DEGs in LF1/LF7

Category	Term	Fold enrichment	FDR
GOTERM_ BP_FAT	GO:0016126~ Sterol biosynthetic process	47.1	5.24E-05
	GO:0006695~ Cholesterol biosynthetic process	43.8	1.49E-03
	GO:1902653~ Secondary alcohol biosynthetic process	42.8	1.60E-03
	GO:0016125~ Sterol metabolic process	27.7	2.35E-06
	GO:0008203~ Cholesterol metabolic process	25.3	5.13E-05
	GO:1902652~ Secondary alcohol metabolic process	24.2	6.36E-05
	GO:0006694~ Steroid biosynthetic process	19.8	1.70E-04
	GO:0008202~ Steroid metabolic process	14.4	1.48E-05
	GO:1901615~ Organic hydroxy compound metabolic process	11.3	1.23E-05
	GO:0044283~ Small molecule biosynthetic process	9.3	5.21E-05

GO terms are indicated in descending order of fold enrichment.

BP, biological process; DEG, differentially expressed genes; FAT, functional annotation tool; FDR, false discovery rate; GO, Gene ontology; LF7, a low-fat diet with pyridoxine hydrochloride (PN) 7 mg/kg diet; LF1, a low-fat diet with PN 1 mg/kg diet

Table S3. Top 10 enriched GO terms of downregulated DEGs in LF1/LF7

Category	Term	Fold enrichment	FDR
GOTERM_ BP_FAT	GO:0035456~ Response to interferon-beta	31.8	4.28E-03
	GO:0034341~ Response to interferon-gamma	18.4	2.44E-03
	GO:0031331~ Positive regulation of cellular catabolic process	11.9	2.23E-03
	GO:0050792~ Regulation of viral process	10.2	1.68E-04
	GO:0043903~ Regulation of symbiosis, encompassing mutualism through parasitism	9.3	3.10E-04
	GO:0009896~ Positive regulation of catabolic process	9.0	7.87E-03
	GO:0043900~ Regulation of multi-organism process	7.0	1.82E-03
	GO:0034097~ Response to cytokine	5.8	6.42E-04
	GO:0045087~ Innate immune response	5.4	2.99E-03
	GO:0002682~ Regulation of immune system process	3.7	3.45E-03

GO terms are indicated in descending order of fold enrichment.

BP, biological process; DEG, differentially expressed genes; FAT, functional annotation tool; FDR, false discovery rate; GO, Gene ontology; LF7, a low-fat diet with pyridoxine hydrochloride (PN) 7 mg/kg diet; LF1, a low-fat diet with PN 1 mg/kg diet

Table S4. Top 10 enriched GO terms of upregulated DEGs in HF1/HF7

Category	Term	Fold enrichment	FDR
GOTERM_ BP_FAT	GO:0009063~ Cellular amino acid catabolic process	50.9	7.97E-04
	GO:0006520~ Cellular amino acid metabolic process	21.0	9.85E-04
	GO:0046395~ Carboxylic acid catabolic process	20.2	1.22E-02
	GO:1901605~ Alpha-amino acid metabolic process	18.4	1.58E-02
	GO:0046394~ Carboxylic acid biosynthetic process	18.0	1.79E-03
	GO:0016054~ Organic acid catabolic process	17.9	1.73E-02
	GO:0016053~ Organic acid biosynthetic process	16.8	2.34E-03
	GO:0044283~ Small molecule biosynthetic process	11.2	1.71E-03
	GO:0019752~ Carboxylic acid metabolic process	6.5	2.14E-02
GOTERM_ MF_FAT	GO:0030170~ Pyridoxal phosphate binding	51.1	1.65E-02

GO terms are indicated in descending order of fold enrichment.

BP, biological process; DEG, differentially expressed genes; FAT, functional annotation tool; FDR, false discovery rate; GO, Gene ontology; MF, molecular function; HF7, a high-fat diet with pyridoxine hydrochloride (PN) 7 mg/kg diet; HF1, a high-fat diet with PN 1 mg/kg diet

Figure S1.

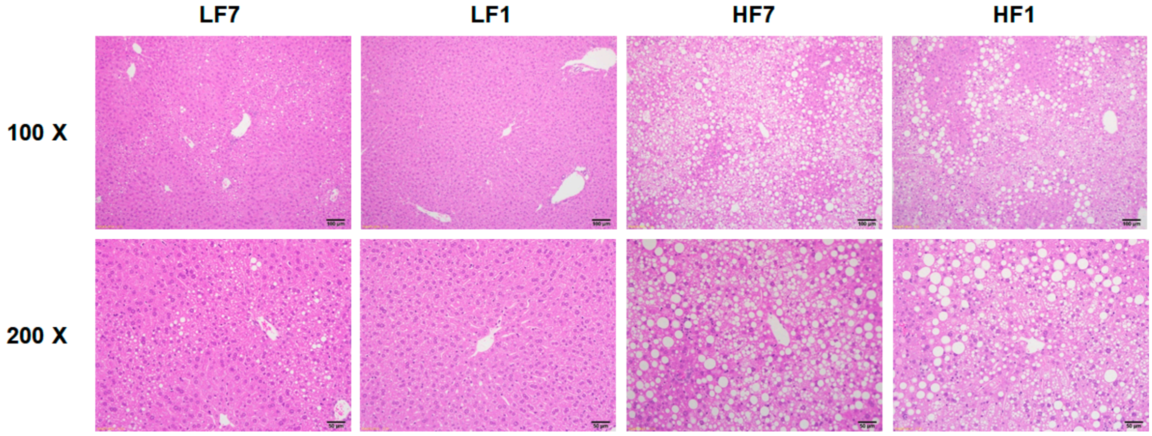

Figure S1. Representative hematoxylin and eosin staining of the liver (n = 4). Upper panel at 100× magnification and lower panel at 200× magnification. LF7, a low-fat diet with pyridoxine hydrochloride (PN) 7 mg/kg diet; LF1, a low-fat diet with PN 1 mg/kg diet; HF7, a high-fat diet with PN 7 mg/kg diet; HF1, a high-fat diet with PN 1 mg/kg diet.
